# Association between labour market trends and trends in young people's mental health in ten European countries 1983-2005

**DOI:** 10.1186/1471-2458-9-325

**Published:** 2009-09-08

**Authors:** Anton CJ Lager, Sven G Bremberg

**Affiliations:** 1 Centre for Health Equity Studies, CHESS, Stockholm University/Karolinska Institutet, Stockholm, Sweden; 2 Department of Public Health Sciences, Karolinska Institutet, Stockholm, Sweden; 3 Swedish National Institute of Public Health, Östersund, Sweden

## Abstract

**Background:**

Mental health problems have become more common among young people over the last twenty years, especially in certain countries. The reasons for this have remained unclear. The hypothesis tested in this study is that national trends in young people's mental health are associated with national trends in young people's labour market.

**Methods:**

National secular changes in the proportion of young people with mental health problems and national secular labour market changes were studied from 1983 to 2005 in Austria, Belgium, Denmark, Finland, Hungary, Norway, Spain, Sweden, Switzerland and the United Kingdom.

**Results:**

The correlation between the national secular changes in the proportion of young people not in the labour force and the national secular changes in proportion of young people with mental health symptoms was 0.77 for boys and 0.92 for girls.

**Conclusion:**

Labour market trends may have contributed to the deteriorating trend in mental health among young people. A true relationship, should other studies confirm it, would be an important aspect to take into account when forming labour market policies or policies concerning the delivery of higher education.

## Background

Several studies indicate that mental health problems have become more common among young people in high-income countries during the last fifty years [1,2]. In a comprehensive review, Rutter et al. did not find any clear explanation for this trend but discussed three main possible causes. Firstly, the transition from dependence on parents to status as young adults has been constantly pushed to a later and later age. Fifty years ago, most adolescents participated in the workforce and were relatively independent of their parents. Today almost all are in education. Their status is similar to children in spite of physically being adults, a situation that might cause strain. Secondly, increasing affluence has given better prospects for most young people. Yet, expectations might have increased at an even more rapid pace, which in turn might lead to frustration. Thirdly, both alcohol and illegal drugs are used more by young people. Such use exacerbates mental health problems.

In Sweden, mental health problems increased at a very high rate during the 1990s. Six independent, population-based studies indicate that the proportion of young people with symptoms like low-spiritedness and being anxious increased two to threefold during this decade [3]. Few other European countries have reported a similar increase during this period. Thus, the development in Sweden enables examination of some of the hypotheses for explaining the current trend in adolescent mental health. A government commission was set up to clarify this issue. As a part of this commission more than 50 potential explanations for the trend were examined: changes in the number of divorces, staff-to-pupil ratios in schools, physical exercise etc. [3]. A specific factor was understood to potentially explain the time trend if two criteria were fulfilled. Firstly, published studies had to present evidence for the effects of the factor on mental health in adolescents. Secondly, the time trend for the factor had to coincide with the development of mental health problems. Only two factors fulfilled both criteria: use of alcohol and the development on the labour market, with decreasing rates of employment, but increasing rates of unemployed and young people not in the labour force, due to schooling or other reasons. Use of alcohol as a risk factor for mental health problems is well established. The remaining factor, labour market changes, is the focus of this paper.

From a societal point of view, unemployment is more problematic among young people than in other age groups since young people lack previous experience of working life. If they remain unemployed, it might be hard for them to take up a job later on. Accordingly, in some high-income countries young people are offered publicly funded jobs instead of receiving unemployment benefits. An even more attractive alternative from the governmental perspective is to offer adolescents longer schooling. This option has two advantages. Firstly, high-income countries need a skilled workforce in order to be able to compete in the world market. Longer schooling will contribute to this. Secondly, to keep adolescents in schools is less costly for the state than to distribute unemployment benefits. Since governments can affect the rate of unemployment by supplying publicly funded jobs and by increasing the length of schooling, it is justified to analyse rates of employment along with rates of unemployment and rates of not being in the work force. Most young people that are not in the work force are studying. Yet, some are not in any organised activity and are not recorded as unemployed. This group has probably increased in high-income countries [4]. However, there are no cross-national time-series on the size of this group.

Getting a job is a major worry for young people [5-10]. Thus, poorer job prospects might affect young people in general. Obviously, young people graduating from school with low grades are especially vulnerable. Yet, even before that, adolescents might be influenced. If job prospects are poor, they know that they have to stay longer in school, regardless of their interest in education. Younger students also know that they have to perform well at school in order to get the grades needed for a job. These phenomena might not be apparent in young adolescents when a job is in the distant future but might appear at the end of compulsory schooling, i.e. around the age of 15 in most high-income countries.

In this study, it was hypothesised that national trends in young people's mental health are associated with national trends in the labour market for young people. To test this hypothesis, the development of adolescent mental health problems in different European countries was compared with the development of the labour market for young people in these countries.

## Methods

### Design

Information on the rate of mental health problems was obtained from the WHO study Health Behaviour in School-aged Children (HBSC) which started in 1983/84 [11]. In this study, similar questions have been posed to nationally representative samples of adolescents in different countries at four-year intervals. Questions on mental health problems might be interpreted differently in different countries, e.g. due to dissimilar cultural attitudes. Therefore it is problematic to compare frequencies. However, if ratios, i.e. relative changes in rates, are studied, these sources of error might mean less. A similar problem is connected with the perception of labour market prospects. If unemployment has been high for a long time in a country, young people might perceive this as the norm and therefore be less mentally affected by the situation. However, this source of error should also mean less if ratios are studied.

### Countries included

There is data from 1985/86 to 2001/02 for twelve countries in the HBSC-database: Austria, Belgium, Denmark, Finland, Hungary, Israel, Norway, Spain, Scotland, Sweden, Switzerland and Wales. Israel is not a member of the OECD and was excluded due to lack of labour force statistics. Specific labour force statistics for Scotland and Wales were not available. For the health data from Scotland and Wales, simple arithmetic averages were therefore calculated and analysed together with labour force statistics for the whole of UK.

### Mental health measures

Rates of mild mental health symptoms were obtained from 15-year-olds in the HBSC study. In HBSC there is also information available for 11- and 13-year-olds but prospects on the labour market were understood to be less relevant for the younger students. There were on average four data-collections per country during the period 1985/86 to 2001/02. In 2001/02, on average 1390 15-year-olds from each country were included [11].

A question in the HBSC that is repeated for different symptoms was used: "In the past 6 months, how often have you...?". Eight symptoms in the HBSC are measured in this way: "...felt low?", "...been in a bad mood (irritable)?", "...felt nervous?", "...felt dizzy?", "...had difficulty getting to sleep?", "...had headache?", "...had stomachache?", and "...had backache?". Three questions, that were thought to be most reliable, were selected for the analysis: feeling low, difficulties getting to sleep, and headache.

The response alternatives were "Rarely or never", "About once every month", "About once every week", "More than once a week" and "About every day". The two last alternatives were collapsed and used as "more than once a week" in the analysis.

There is mental health data for 1985/86, 1993/94, 1997/98 and 2001/02 in the HBSC-database. For Austria, Finland and Norway there is also information on 1983/84. Information on 1985/86 is missing for Finland and Denmark. For Belgium, all health data is collected only in the French-speaking parts. For Hungary, there were no labour force statistics available prior to 1992. Hungary is therefore included in the analysis from 1993/94. For Spain there is information from 1985/86 and 2001/02, but not from the collections in between. Information on 1993/94 is missing for Switzerland and there is no information on difficulty in getting to sleep from Switzerland.

For two countries, the international HBSC-data was supplemented with data published in national reports: Austria for 1989/90 and Sweden for 2005/06 [12,13].

### Labour market measures

Yearly data on the proportion of 15-24-year-olds that were employed, unemployed, and not in the labour force was obtained for each country from OECD's Labour Force Statistics (LFS) (available at ). Labour force statistics from OECD were supplemented with national data for Austria between 1983-1993 and Switzerland in 1980 [14] (E. Grisafi Favre, personal communication, February 10, 2006). The definition of unemployment used in the Austrian report generates double the proportion compared with the OECD-definition for 1994-1997. It was assumed that this also holds for the period before 1994 in Austria.

### Statistical methods

The main statistical approach was to test if the mental health and labour market trends were correlated. This was done in the following manner: First, to be able to use as much of the available information as possible, linear regressions were fitted to give a corrected estimate of the rate at the earliest year, typically 1983 or 1985, and latest year, typically 2002, available for each country. This procedure was used for both the HBSC data on symptoms (from, on average, four years per country) and the labour market data from the OECD (typically available for each intermediate year), with the exception for the health data from Spain where the only available HBSC data, from 1985/86 and 2001/2002, were used directly.

Then symptom-specific relative changes in the proportion with problems were calculated by dividing the rates estimated from the regression for the latest and earliest years available for each specific country. A mean relative change in proportion with symptoms was also calculated, by taking the arithmetic mean of the three symptom-specific ratios. Switzerland lacked data on problems with getting to sleep. To be able to calculate a mean relative change in proportion with symptoms for Switzerland, the arithmetic mean of the ratios in problems with getting to sleep in the rest of the sample was imputed: 1.10 for boys and 1.31 for girls. A relative change in the proportion of young people in each labour market group was calculated by dividing the corrected estimate for the latest available year with the earliest. Here, men and women were treated together to keep the analysis simple and to keep down the total number of correlations.

Finally, Pearson's correlations were calculated for changes in each of the three labour market groups versus each of the three symptoms plus the mean symptom change, for boys and girls separately, giving a total of 3*(3+1)*2 = 24 correlations. Confidence intervals at the 95%-level were calculated using Fisher's z'-scores for two-tailed tests.

The rates at the earliest and latest year were corrected before they were used in the main analysis. Such corrections could lead to biased standard errors. To avoid this problem and still be able to use all the available information, a panel regression was also fitted to the original data, with fixed effects for countries.

## Results

Information from ten European countries was analysed for the period 1983/85 to 2002. During this period, the proportion of 15-year-old boys with mental health symptoms varied from 2.2 to 21.2 percent. The proportion of 15-year-old girls with mental health symptoms varied from 5.6 to 30.4 percent, see Table 1. Mental health problems became more common during the period. The proportion of 15-to-24-year-olds that were employed varied between 26.10 and 66.26. Employment rates decreased, and the proportion not in the labour force increased, during the period, while unemployment rates varied.

**Table 1:** Proportions (percentage) of 15-year-olds feeling low, having difficulties getting to sleep and headache and proportions of 15-24-year-olds employed, unemployed and not in labour force, in ten countries in the earliest (typically 1985) and latest year available (typically 2002).

**Country**	**Year**	**Feeling low**		**Difficulties getting to sleep**		**Headache**		**Labour market status**		
		**Boys**	**Girls**	**Boys**	**Girls**	**Boys**	**Girls**	**Employed**	**Unemployed**	**Not in labour force**
Austria	1983	2.2	5.6	8.9	14.5	3.1	11.1	61.2	2.8	35.8
	2002	3.6	7.5	8.7	15.1	8.2	22.4	53.4	3.2	43.4
										
Belgium	1985	10.7	14.2	14.9	20.0	6.2	13.3	32.3	7.9	59.9
	2002	13.0	21.4	12.4	20.0	11.6	23.0	26.1	5.5	68.4
										
Denmark	1993	1.8	13.8	12.3	15.2	3.8	10.5	63.8	8.6	27.6
	2002	4.8	16.8	14.3	18.2	4.8	13.5	65.7	4.6	29.7
										
Finland	1983	6.7	12.4	14.4	18.0	2.6	11.5	50.0	5.3	44.7
	2002	7.3	15.6	11.6	15.9	8.8	25.2	32.4	12.9	54.8
										
Hungary	1993	17.0	30.5	11.0	12.2	8.2	24.7	31.9	8.6	59.5
	2002	19.0	31.5	11.0	21.2	11.2	30.7	31.8	3.5	64.8
										
Norway	1983	6.0	10.9	10.3	14.4	4.4	13.6	56.4	4.9	38.7
	2002	7.6	19.3	12.5	18.5	8.1	20.6	53.9	7.5	38.6
										
Spain^a^	1985	9.2	13.9	9.7	18.4	9.7	17.7	33.4	23.4	43.3
	2002	14.6	29.1	11.9	20.9	14.1	28.9	33.4	12.0	54.7
										
Sweden	1985	4.7	12.2	14.9	15.8	4.8	16.1	63.1	4.7	32.2
	2005	14.4	35.6	21.2	30.4	13.0	30.8	38.0	10.1	51.9
										
Switzerland	1985	9.0	23.8	-	-	5.7	16.1	67.1	1.8	31.1
	2002	6.6	21.6	12.3	21.0	6.5	17.6	62.6	4.4	33.0
										
UK	1985	6.6	8.3	14.3	18.2	4.8	11.7	66.3	12.3	21.4
	2002	9.8	21.6	17.9	27.5	12.1	28.5	59.4	7.8	32.8

Note: All proportions are corrected estimates resulting from linear regressions, see "Statistical methods" section. ^a^For Spain, the table gives the actual observed proportions with symptoms.

**Figure 1:**
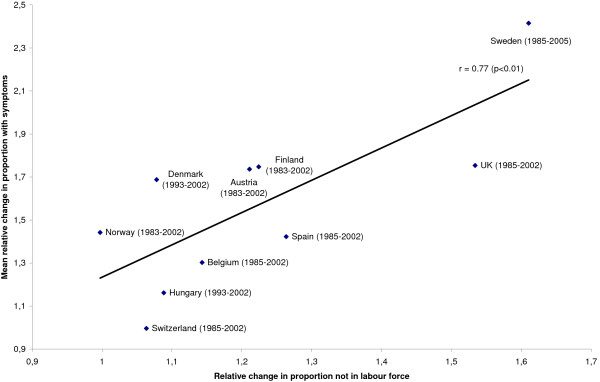
**Relative change in proportion 15-24-year olds not in labour force and mean relative change in proportion 15-year-old boys with symptoms**. The years included for each country are shown in brackets.

**Figure 2:**
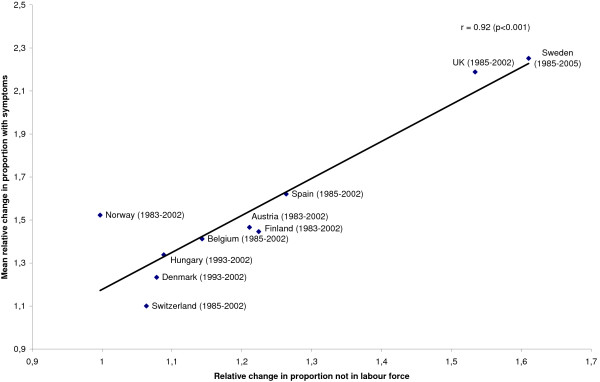
**Relative change in proportion 15-24-year olds not in labour force and mean relative change in proportion 15-year-old girls with symptoms**. The years included for each country are shown in brackets.

Changes in the proportion of 15-to-24-year-olds not in the labour force were significantly associated with mean changes in the proportion of 15-year-olds with mental health symptoms, both in boys, see Figure 1, and in girls, see Figure 2. For boys, 59 percent of the cross-national variations in secular changes of mental health symptoms were accounted for by changes in young people not in the labour force. For girls, 85 percent of the variation was accounted for.

Pearson's correlations between changes of rates of specific mental health problems and specific labour market positions are given in Table 2. The panel regressions produced similar results with two exceptions: changes in the proportion employed did not significantly predict mean changes in the proportion of boys with symptoms, while changes in the proportion not in labour force did predict changes in the proportion of boys feeling low, see Table 3.

**Table 2:** The association between changes of rates of mental health problems and labour market position in ten European countries: Pearson's correlations.

	**Feeling low**		**Difficulties getting to sleep**		**Headache**		**Mean change**	
	**Boys**	**Girls**	**Boys**	**Girls**	**Boys**	**Girls**	**Boys**	**Girls**
Employed	-0.26	-0.39	0.08	-0.08	-0.81**	-0.58^a^	-0.65*	-0.48
Unemployed	-0.06	-0.02	0.00	-0.02	0.43	0.08	0.23	0.02
Not in labour force	0.52	0.86**	0.52	0.49	0.58^a^	0.70*	0.77**	0.92***

*p < 0.05, **p < 0.01, ***p < 0.001, ^a^p < 0.10

**Table 3:** The association between changes of rates of mental health problems and labour market position in ten European countries: coefficients estimated from panel regression.

	**Feeling low**		**Difficulties getting to sleep**		**Headache**		**Mean change**	
	**Boys**	**Girls**	**Boys**	**Girls**	**Boys**	**Girls**	**Boys**	**Girls**
Employed	-0.14	-0.26	0.05	0.03	-0.20^a^	-0.46*	-0.10	-0.23
Unemployed	-0.16	-0.45	-0.12	-0.31	-0.08	-0.23	-0.12	-0.34
Not in labour force	0.30**	0.62**	-0.01	0.12	0.33**	0.81***	0.21*	0.52**

*p < 0.05, **p < 0.01, ***p < 0.001, ^a^p < 0.10

## Discussion

In this preliminary study, there is an association between changes in the proportion of 15-year-olds with symptoms (feeling low, headache and difficulties getting to sleep) and changes in the proportion of 15-24-year-olds not in the labour force. This suggests that changes in the national labour market situation for young people might have contributed to the deteriorating trends in mental health and that further research in this new area is motivated.

The study is based on aggregated data, and such data is often disregarded due to the ecological fallacy [15,16]. The ecological fallacy means making assumptions about individual level correlations on the basis of aggregated data. At the same time, variables at this aggregated level are interesting since they are possible to influence by policies or interventions at the national level. Such interventions are strictly not dependent on the exact mechanisms involved. However, they are based on the assumption that there is a true relationship. Therefore, this preliminary study needs to be replicated. Should the association be confirmed, a natural next step would then be to try to identify the mechanisms involved (e.g. worries or changes in educational climate). To avoid the ecological fallacy, data on such mechanisms should be at the individual level.

A problem in this study is the risk of possible confounding. Yet, since the analysis focus changes over time, the confounders must be factors associated to both changes in mental health problems and changes in labour market trends - at the same time. Such confounders are possible but the risk seems lower than in ordinary cross-sectional country comparisons. Still, since the number of countries in this study is low, it is crucial that the analysis is replicated. Further, although the tests suggest that the associations detected in this study are not due to chance, it is possible that unknown systematic errors could explain the association.

A replication will be possible with data stemming from more and more countries joining the HBSC-network. With more power in the study, possible confounders (e.g. changes in population composition) and mediators (e.g. changes in educational climate) could be taken into account. A true relationship, should other studies confirm it, would be an important aspect to take into account when forming labour market policies or policies concerning the delivery of higher education.

## Conclusion

Labour market trends may have contributed to the deteriorating trend in mental health among young people. A true relationship, should other studies confirm it, would be an important aspect to take into account when forming labour market policies or policies concerning the delivery of higher education.

## Competing interests

The authors declare that they have no competing interests.

## Authors' contributions

The paper was based on an idea by AL and SB, drafted by AL and revised by SB. Both authors read and approved the final version of the manuscript.

## Pre-publication history

The pre-publication history for this paper can be accessed here:


